# Ophiopogon Saponin C1 Inhibits Lung Tumors by Stabilizing Endothelium Permeability via Inhibition of PKCδ

**DOI:** 10.7150/ijbs.34978

**Published:** 2020-01-01

**Authors:** Yuanyuan Zhang, Yazheng Zhao, Yan Wu, Jin Qi, Fang Li, Junping Kou, Boyang Yu

**Affiliations:** Jiangsu Key Laboratory of TCM Evaluation and Translational Research, School of Traditional Chinese Pharmacy, China Pharmaceutical University, 639 Longmian Road, Nanjing 211198, China.

**Keywords:** Ophiopogon Saponin C1, Endothelial Cell Permeability, Lung Cancer, TNF-α, PKCδ

## Abstract

As the most frequent cause of cancer-related death worldwide, lung cancer is closely related to inflammation. The interaction between tumor cells and inflammatory cells promotes tumor development and metastasis. During tumor development, vascular endothelial cells form the most important barrier to prevent tumor cell migration to the blood and tissue. Increased vascular permeability provides favorable conditions for the migration of tumor cells, and endothelial tight junctions are an important component of the vascular barrier. Protein kinase C δ is involved in the occurrence of non-small cell lung cancer and regulates vascular permeability and tight junction protein expression. Src kinase was reported to play an important role in TNF-α-induced endothelial inflammation. Ophiopogon Saponin C1 is a new chemical compound isolated from *Liriope muscari*, but its pharmacological activities have not been fully elucidated. Therefore, we tested the protective effects of C1 on endothelial permeability in a model of TNF-α-induced endothelial inflammation by transendothelial electrical resistance and sodium fluorescein assays and verified these results in a nude mouse model of experimental pulmonary adenocarcinoma metastasis. We further elucidated the mechanism of C1, which was based on the PKCδ and Src proteins, by Western blotting. C1 can inhibit lung cancer in vivo, regulate the level of plasma inflammation in tumor-bearing mice, and protect the pulmonary vascular barrier against injury induced by cancer. It was investigated the expression and distribution of the TJ index protein ZO-1 in mouse vascular endothelium and HUVECs and found that C1 could inhibit the degradation and breakage of the ZO-1 protein. Related signaling experiments confirmed that C1 can inhibit TNF-α and activation of PKCδ and Src kinase. This study laid the foundation for further analysis of new drugs with clear mechanisms and independent intellectual property rights of traditional Chinese medicines.

## Introduction

Malignant neoplastic disease is one of the most common causes of mortality worldwide and has a high rate of death. Among cancers, lung cancer is the most frequent cause of cancer-related death worldwide. Every year, 1.8 million people are diagnosed with lung cancer, and 1.6 million people die due to this disease. The 5-year survival rates vary from 4 to 17% depending on the stage and regional differences 1. Clinical data shown that 80% of cancer patients died because of tumor metastasis, and thus, control of tumor metastasis is a major strategy for cancer treatment 2. Age is the single most significant risk factor for cancer development, with the majority of cancer cases being diagnosed after the age of 65 3. Aging is a biological process that occurs in virtually all organisms and is characterized by a progressive loss of organ function and decline in tissue renewal capacity 4-6.

Endothelial cells play an important role in tumor metastasis 7. During tumor metastasis, vascular endothelial cells form the most important barrier to prevent tumor cell migration to the blood and tissue 8. Increased vascular permeability provides favorable conditions for the migration of tumor cells, and endothelial tight junctions (TJs) are an important factor in the vascular barrier. Aging is an important risk factor for the progressive decline of endothelial function 9. Vascular endothelial dysfunction occurs during the human aging process and is accompanied by deterioration in the balance between vasodilator and vasoconstriction substances produced by the endothelium 10, 11. Therefore, by protecting endothelial cell integrity, maintaining TJs of endothelial cells, and inhibiting invasion and metastasis of lung cancer cells, effective intervention in the early stage of lung cancer occurrence and metastasis will be an important direction for future research and development of anti-lung cancer metastasis drugs.

ZO-1 is an important cytoplasmic protein that is also a major component of TJs. This protein connects to the main cell body to act as an intermediate transmembrane protein and part of the actin cytoskeleton. Functional and structural changes can cause the dissociation of TJs and an increase in the intercellular space, thereby increasing the vascular permeability 12, 13. ZO-1 expression is regulated by a variety of signaling pathways. Previous studies have shown that MAPK, MLC, Src and PKC are involved in the regulation of ZO-1 protein expression 14-16. Early laboratory studies shown that activation of Src in vascular endothelial cells could activate the PI3K/Akt signaling pathway and mediate the degradation of ZO-1 17. Clinical studies reported that PKCδ is highly expressed in the lung epithelial tissues of non-small cell lung cancer (NSCLC) patients, and its inhibitor rotterlin significantly induced tumor apoptosis, indicating that PKCδ is involved in the occurrence of NSCLC 18. Other studies have shown that PKCδ has a role in inflammatory factor-mediated endothelial inflammatory models 19. PKCδ can be activated in a Src-dependent manner 20. Src regulation of PKCδ has also been reported 21. However, PKCδ has little effect on Src, and this interaction has not been reported in vascular endothelial cells.

Our lab extracted the steroidal saponin DT-13 and the aglycone Ruscogin, which were shown to have good anti-inflammatory and anti-tumor activities, from *Ophiopogon japonicas* 22,23. Ophiopogon Saponin C1 (C1) is a new compound isolated from the roots of *Liriope muscari* (Decne.) L.H. Bailey, but its pharmacological effects have not been reported 24. As to the traditional Chinese medicine theory, the *Liriope muscar* was used for lung disease. Therefore, in this study, the anti-lung tumor effect of C1 was determined in a nude mouse model of experimental pulmonary adenocarcinoma metastasis, and its mechanism based on the proteins PKCδ and Src was further elucidated. This study laid foundation for further analysis of new drugs with clear mechanisms and independent intellectual property rights of traditional Chinese medicines.

## Materials and Methods

### Extraction and isolation of C1

C1 was prepared as previously described and identified as 25(R)-ruscogenin-1-O-[β-d-glucopyranosyl-(1→2)][β-d-xylopyranosyl-(1→3)]-β-d-fucopyranoside by comparison of its physical data (^1^H NMR, ^13^C NMR, MS) with published values. The purity was shown to be 98.5% using HPLC-ELSD assays as previously reported 24.

### Cell culture

HUVECs and A549 cells were purchased from the Shanghai Institute of Cell Biology, Chinese Academy of Sciences. Cells were grown in RPMI 1640 medium (Invitrogen Life Technologies, Carlsbad, CA, USA) supplemented with 10% heat-inactivated fetal bovine serum (FBS, ScienCell, CA, USA), 100 U/mL penicillin, 100 μg/mL streptomycin and 2.0 g/L sodium bicarbonate. Cells were maintained at 37°C with 5% CO_2_ and 95% humidity.

### Transendothelial electrical resistance (TEER) assays and sodium fluorescein (Na-F) assays

HUVECs were seeded on transwell inserts (0.4 μM pore, 6.5 mm diameter, Millipore, USA) for 7 days. The TEER of the monolayer was also measured daily with a Millicell-ERS voltohmmeter (Millipore, USA). Resistance values of multiple transwell inserts of an experimental group were measured sequentially, and the mean was expressed in the common unit (Ω·cm^2^) after subtraction of the value of a blank cell-free filter. The TEER of the monolayers was recorded when a stable resistance reading was achieved with triplicate measurements that were taken for each transwell. C1 (0.01-1 μM) was added to the upper chamber for 1 h, and 10 ng/mL TNF-α (Bioworld, USA) was added for 4 h. Paracellular permeability was assessed by the addition of Krebs-Ringer buffer (118 mM NaCl, 4.7 mM KCl, 1.3 mM CaCl_2_, 1.2 mM MgCl_2_, 1.0 mM NaH_2_PO_4_, 25 mM NaHCO_3_, and 11 mM D-glucose, pH 7.4) containing 100 μg/mL Na-F to the top chamber. The fluorescence was measured after 30 min at 37°C. The Na-F concentration was determined using a fluorescence multiwall plate reader [Ex (λ) 485 nm; Em (λ) 530 nm; Thermo].

### Co-culture of A549 and HUVECs

The 10 μg/mL fibronectin solution was added at 100 μL/well in an 8 μm Millicell chamber and incubated at 4°C overnight (Cat#: PIEP12R48). The well was pretreated with cold PBS 2~3 times. Then, 200 μL of HUVECs in the logarithmic growth phase was added in the inner chamber with 1 mL RPMI 1640 complete medium in the outer chamber. The next day, the medium was changed. After 7 days of continuous culture, C1 (0.01-1 μM) was added to the upper chamber for 1 h, and 10 ng/mL TNF-α (Bioworld, USA) was added for 4 h. A549 cells in logarithmic growth phase were pretreated by serum-free medium RPMI 1640 for 1 h, and then, the cells were collected and labeled with 1 μΜ Calcein-AM for 15 min. The samples were added to a small inner chamber, and 1 mL RPMI 1640 complete medium was added. After 48 h, the chamber was removed, the cells were carefully wiped out the chamber, and 4% polyformaldehyde was used to fix the cells at the bottom of the compartment. Then, the chamber with the cells was dried. The migrating cells were observed under a fluorescence microscope.

### Animals and experimental design

Ten-week-old male nude mice were obtained from Yangzhou University (Yangzhou, China). Mice were housed in microisolator cages in a pathogen-free facility. During the logarithmic growth phase, A549 cells were washed 2 times after routine digestion. A 200-cell mesh filter was used to obtain a single cell suspension. The cell concentration was 10^7^ cells/mL in serum-free DMEM with PBS. The nude mice were inoculated; the tail was soaked for 2 min at 45°C to expand the tail vein, and the A549 single cell suspension was slowly and uniformly injected at a concentration of 10^6^ cells/0.1 mL serum-free DMEM into the tail vein of the nude mice. The mice were weighted after the second day of inoculation. Except for the nude mice in the control group, the other nude mice were randomly divided into 3 groups: the model group, the C1 administration group and the topotecan hydrochloride (TPT) positive control group. The control group and the model group were treated every day by intragastric administration of 0.5% 10 g/0.1 mL CMC-Na, and the drug group was administered 4 mg/kg C1 every day by intragastric administration (0.4 mg/mL, 10 g/0.1 mL, CMC-Na 0.5%, dissolved). The positive control group was intravenously injected with 2 mg/kg TPT (0.2 mg/mL, 10 g/0.1 mL, CMC-Na 0.5% dissolved) 2 times a week. After the fourth week of inoculation, the model was established. The lung tissue and blood samples were collected, and serum was frozen at -80°C for ELISAs.

### Immunofluorescence staining

The immunofluorescence staining of ZO-1 in vivo was performed as described previously with a few modifications 25. Aortas from the mice were rapidly excised under general anesthesia, carefully trimmed to remove fat and connective tissue and washed twice by ice-cold PBS. Then, the aortas were opened longitudinally to expose the endothelium and pinned onto 4% agar. HUVECs were cultured to confluence on glass cover slips in complete media containing 10% FBS and maintained for 7 days. Cells were then stimulated with TNF-α (10 ng/mL) for 4 h with or without prior treatment with C1 (1 μM) for 1 h. The aortas or cells were washed with PBS, fixed in 4% formaldehyde in PBS (v/v) for 30 min at room temperature, and permeabilized in 0.1% Triton X-100 in 5% bovine serum albumin (BSA, diluted in PBS) for 30 min at room temperature. The aortas or cells were then blocked with 5% BSA for 1 h at room temperature. Then, they were incubated with rabbit anti-ZO-1 polyclonal antibody overnight at 4°C and washed with PBS three times followed by incubation with donkey anti-rabbit IgG 488-conjugated secondary antibody for 1 h. All samples were assessed using a fluorescence microscope (LSM700, Zeiss, Germany).

### Leakage detection of pulmonary vascular permeability in nude mice

First, 5 mg/mL EBA solution (10 g/0.1 mL) was injected into the tail vein in nude mice after establishment of the model, and the mice were anesthetized after 2 h. Then, 5 mM EDTA-PBS solution was used for right ventricular lavage to remove the dye in the blood vessels. The lung tissue was removed and added to filter paper to dry and weigh. Next, 1 mL formamide solution homogenate was added to 100 mg lung tissue with liquid nitrogen for quick freezing. The homogenate was transferred into a 1.5 mL EP tube, and after incubation for 18 h at 60°C, the samples was centrifuged at 5000 g for 30 min. The supernatant was assessed at 620 nm and 740 nm. At the same time, the -OD value standard curve of EBA was fitted to calculate the EBA content in each lung tissue.

### Western blotting

HUVECs were treated with various concentrations (0.01, 0.1 and 1 µM) of C1 for 1 h following TNF-α (10 ng/mL) stimulation for 4 h. After the cells were washed with PBS, they were lysed using lysis buffer (containing 20 mM Tris, pH 7.5, 150 mM NaCl, 1% Triton X-100, sodium pyrophosphate, β-glycerophosphate, EDTA, Na_3_VO_4_, leupeptin) for 30 min on ice. Protein concentration was measured using a BCA protein assay kit. Equal amounts of protein (40 μg) were separated using SDS-PAGE and then transferred onto a PVDF membrane. The membrane was then blocked with 3% BSA for 1.5 h, followed by overnight incubation at 4˚C in the primary antibody. After the membrane was washed, a HRP-conjugated secondary antibody was added and incubated for 1.5 h. The bands were detected by an ECL kit and quantified by Quantity One software.

### Immunohistochemistry

Frozen sections were washed with PBS for 5 min. The primary antibodies we used were anti-mouse p-PKCδ (1:200 dilution; Abcam), anti-p-SrcTyr416 (1:200; Abcam). Primary antibodies were incubated with tissue sections for 60 min at room temperature. Primary antibodies were diluted in solution (1% bovine serum albumin in 0.01 M TBS, pH 7.2). Then, slides were washed with PBS thrice for 5-min each, followed by incubation with biotin-conjugated corresponding secondary antibodies for 20 min at 37℃. Then, slides were washed with PBS thrice for 5-min each to remove unbound antibody. Samples were incubated with streptococcal avidin-biotin complex reagent for 20 min at 37℃. 3,3'-diaminobenzidine was used for color development (Beyotime, Jiangsu, China). Sections were re-stained with hematoxylin. After dehydration and sealing, sections were observed under a microscope.

### Statistical analysis

GraphPad Prism software (Version 4.0, GraphPad Software Inc., San Diego, CA) was used to perform the data analysis. Data are expressed as the mean±SD. Statistical significance between different groups was calculated by a one-way ANOVA where appropriate or a Student's two tailed t-test; *P* values below 0.05 were considered statistically significant.

## Results

### Effect of C1 on endothelial monolayer permeability and ultrastructural characteristics of TJs in vitro

**Figure 1:**
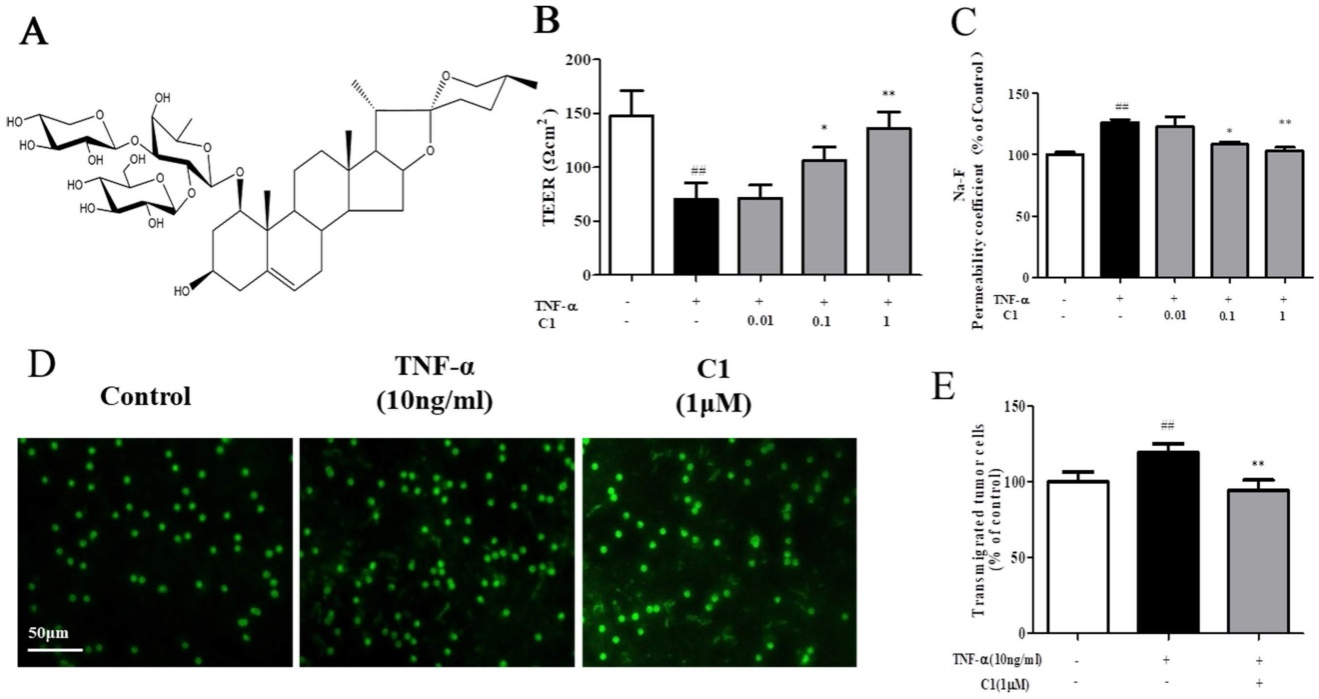
** C1 ameliorated TNF-α-induced endothelial hyperpermeability** and **transendothelial migration of A549 cells.** EC permeability was measured using a Millicell-ERS voltohmmeter. HUVECs were pretreated with various concentrations of C1 (0.01, 0.1 and 1 µM) for 1 h followed by TNF-α (10 ng/mL) stimulation for 4 h. **(A)** The chemical structure of C1. **(B)** The transendothelial permeability was assessed by TEER. **(C)** The transendothelial permeability was assessed using the paracellular transport marker (Na-F) permeability coefficient, which was measured using a fluorescence multiwall plate reader [Ex (λ) 485 nm; Em (λ) 530 nm]. **(D)&(E)** HUVECs grown to confluence on transwell inserts were activated or not (control) with TNF-a (10 ng/mL). After 4 h, calcein-AM-labeled A549 cells were seeded onto the inserts. After 48 h, melanoma cell migration across HUVEC monolayers was analyzed. Bar=50 μm. The data represent the mean±SD from three experiments. ##*P*<0.01 vs. the control group; *P<0.05, **P<0.01 νs. the TNF-α group.

The structure of C1 is shown in Fig. 1A. The effects of TNF-α or C1 on HUVEC monolayer permeability were examined by detecting the TEER of the endothelial cell monolayer and performing transwell Na-F assays. C1 at concentrations of 0.01, 0.1, and 1 μM was applied for 1 h, followed by TNF-α stimulation for 4 h. TNF-α-induced barrier disruption resulted in a sharp decrease in TEER and an increase in the Na-F permeability coefficient. However, C1 enhanced TEER and decreased the Na-F permeability coefficient (Fig. 1B & C). An in vitro co-culture model was established by using HUVECs-A549 cells, and the anti-metastasis effect of C1 was assessed. As shown in Fig. 1D, in a TNF-α-stimulated HUVEC inflammation model, transendothelial migration of A549 cells increased significantly, while 1μM C1 inhibited the migration of A549 cells.

### Effect of C1 on lung cancer in nude mice

**Figure 2:**
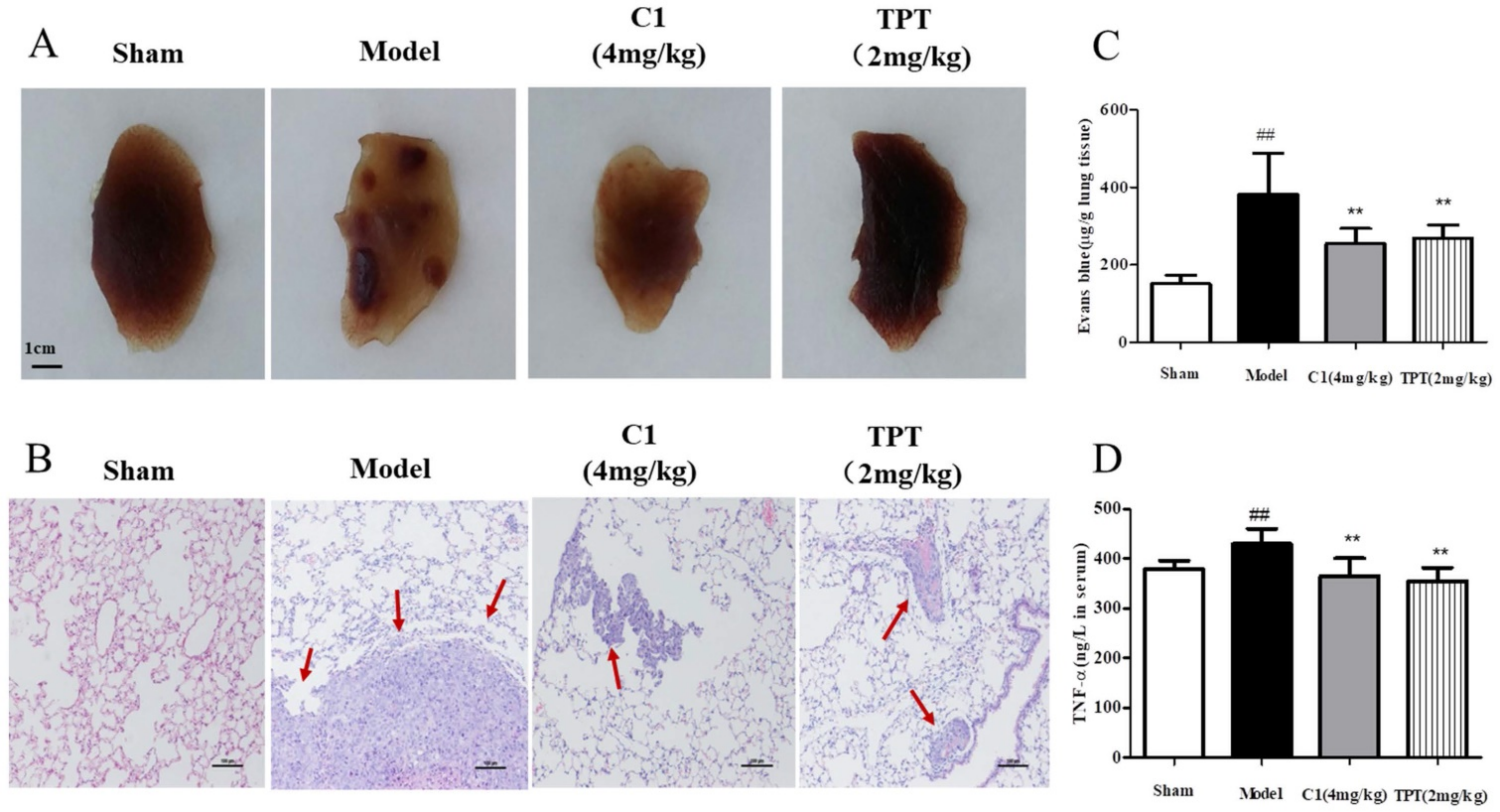
** C1 suppressed lung cancer in the nude mouse model of experimental pulmonary adenocarcinoma metastasis. (A) & (B)** At autopsy, the lungs were removed and fixed in 4% formaldehyde before they were embedded in paraffin, and then, sections were photographed and stained with hematoxylin and eosin. **(C)** Lung vascular permeability was detected using Evans blue leakage after the model was established, and the degree of vascular permeability was reflected by OD values of Evans blue. **(D)** Plasma TNF-α levels of each group were detected with an ELISA kit after model establishment. ^##^*P*<0.01 vs. Sham group; ^**^ *P*<0.01 νs. Model group.

After 4 weeks of inoculation of lung cancer cells in the caudal vein, no death was observed in the model nude mice. After the model was established, the fixed pulmonary tissue images and hematoxylin and eosin staining results, as shown in Fig. 2A, demonstrated that the sham group of nude mice had smooth lung tissues, no tumor nodules, normal tissue distribution, and no cell aggregation; mice in the model group shown multiple visible lung tumor nodules with increased sizes and tissue metastasis, and the cell structures were round or oval, with large nuclei, deep staining, and a nucleocytoplasmic ratio consistent with the pathological features of adenocarcinoma. In contrast, C1 at a dose of 10 g/0.1 mL resulted in no visible lung tumor nodules and significantly decreased lung metastases, with significant inhibition of lung cancer metastasis. The positive control group (TPT treated group) shown similar results.

Next, we demonstrated the in vitro anti-lung cancer effects of C1 on endothelial cell migration by inhibiting TNF-α-induced HUVEC damage in the endothelial barrier. We also confirmed these effects in an experimental model of lung cancer metastasis and on pulmonary vascular tumor cells from the circulation into lung tissues, which act as a barrier. Therefore, we used Evans blue leakage to detect the pulmonary vascular permeability in nude mice treated with C1 to assess experimental metastasis of lung cancer; the experimental results are shown in Fig. 2C. The model group mice shown significantly increased pulmonary vascular leakage of Evans blue, which was approximately 2-3 times higher than that of the normal group, while C1 at a dose of 10 g/0.1 mL significantly inhibited pulmonary vascular leakage of Evans blue and had a protective effect on vascular integrity; the positive control group shown similar results.

Finally, an experimental lung cancer metastasis model was used, which was not the same as the TNF-α-induced HUVEC in vitro model. The occurrence and development of tumors are closely related to their secretion of various inflammatory factors. Therefore, the level of plasma TNF-α in nude mice was detected by ELISA kits, and the results are shown in Fig. 2D. The plasma level of TNF-α in nude mice increased significantly compared with that in the blank control group, while C1 inhibited the increase of TNF-α level in nude mice and regulated the level of inflammation in the tumor-bearing mice. The positive control group also had a significant increase in the level of TNF-α.

### Effect of C1 on the organization and expression of ZO-1 in vitro and in vivo

**Figure 3:**
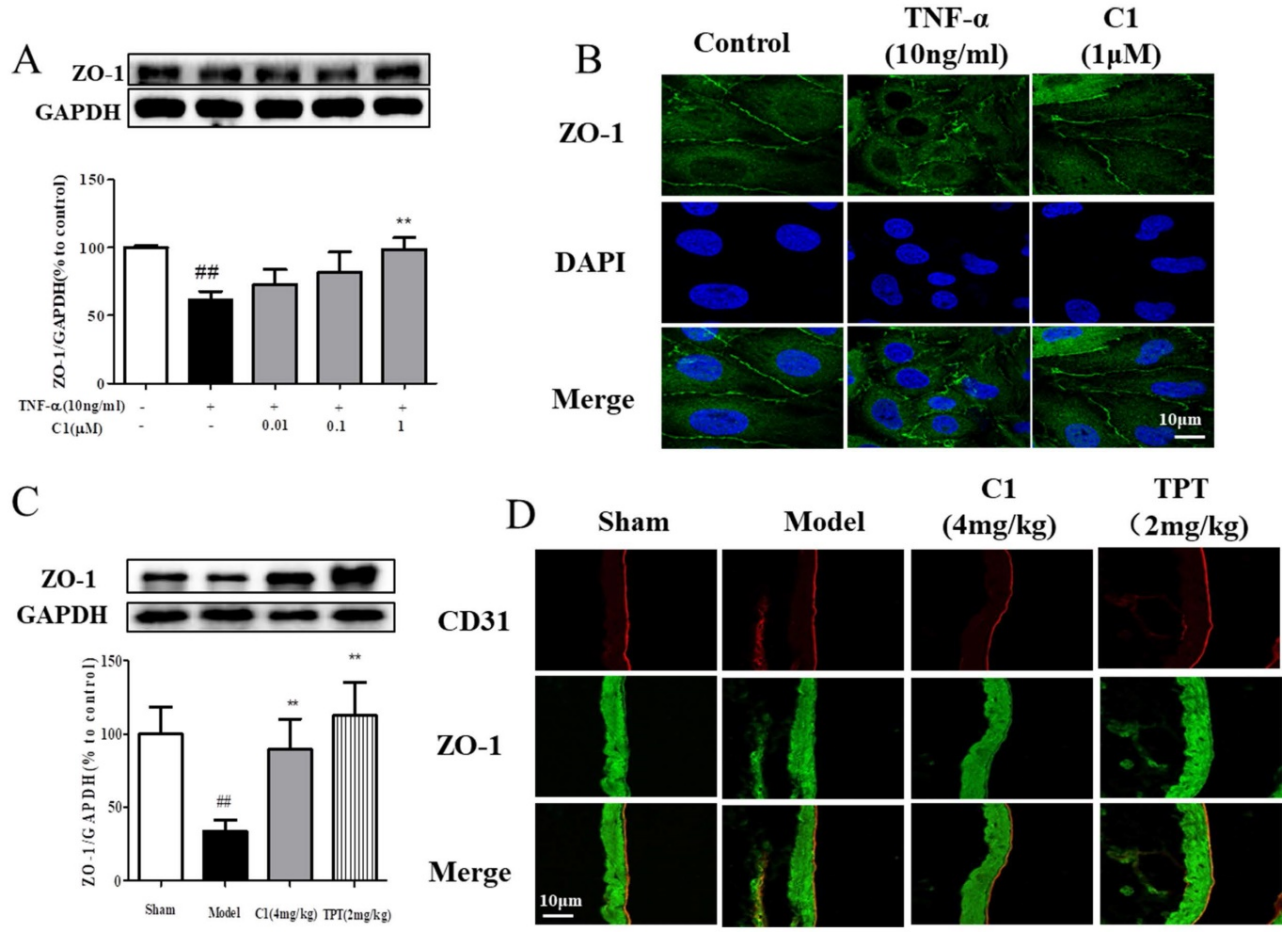
** C1 inhibited degradation and rearrangement of the tight junction protein ZO-1 in vitro and in vivo. (A)** Western blotting analysis revealed that C1 at 1 μM inhibited degradation of the tight junction protein ZO-1 in HUVECs. **(B)** Immunofluorescence analysis shown consistent ZO-1 localization in HUVECs. The data represent the mean±SD from three experiments. ^##^ *P*<0.01 vs. Control group; ^**^P<0.01 νs. the TNF-α group. **(C)** The expression of ZO-1 in mouse lung tissue was detected by Western blotting. **(D)** The distribution of ZO-1 in mouse pulmonary vessel was measured by immunohistochemistry. The results were obtained from three independent experiments and are expressed as the mean±SD. Representative confocal microscopy images of immunostaining of ZO-1 in the mouse pulmonary vessel. ^##^*P*<0.01 vs. Sham group; ^**^P<0.01 νs. Model group.

Downregulation of the TJ protein ZO-1 may be critical for disruption of the endothelial barrier 26. This protein shown discrete localization in response to TNF-α treatment; however, ZO-1 in HUVECs treated with C1 was intact, in contrast to that in the model group. HUVECs were exposed to TNF-α and assessed for ZO-1 expression by Western blot analyses (Fig. 3A). The ZO-1 protein maintains the structural integrity of TJs in endothelial cells. Using immunofluorescence, we identified the localization of ZO-1 at the endothelial cell contacts of confluent cells (Fig. 3B). As shown in Fig. 3C, mice were pretreated with C1 (4.0 mg/kg) for 1 h and then administered TNF-α (100 μg/kg) for 4 h. The ZO-1 protein expression was significantly increased compared to that of the model group. Immunostaining of ZO-1 in the mouse aortic endothelium shown that TNF-α abolished the endothelial junction proteins at the cell-cell contact zones between adjacent cells. C1 (4.0 mg/kg) inhibited ZO-1 disassembly (Fig. 3C).

First, the distribution and expression of ZO-1 in pulmonary vascular endothelial cells were examined by immunofluorescence. Second, Western blotting was used to detect ZO-1 protein expression in lung tissues, and the results are shown in Fig. 3. Immunofluorescence results demonstrated that ZO-1 expression on the endothelial cells of the blank group was continuous, while the ZO-1 on the endothelial cells in the model group appeared discontinuous and filamentous. There was a deletion or breakpoint in the middle of the protein expression. Treatment with 10 g/0.1 mL C1 inhibited the rupture of ZO-1 and maintained a continuous linear shape of the protein expression. Western blotting results shown that ZO-1 expression in the lung tissue of nude mice was significantly decreased by tumor inoculation, while C1 significantly inhibited ZO-1 degradation and maintained its protein expression, which was consistent with the immunofluorescence results. The positive control group also shown the same results (Fig. 3C & D).

### The PKCδ inhibitor rotterlin suppresses the metastasis of A549 cells

**Figure 4:**
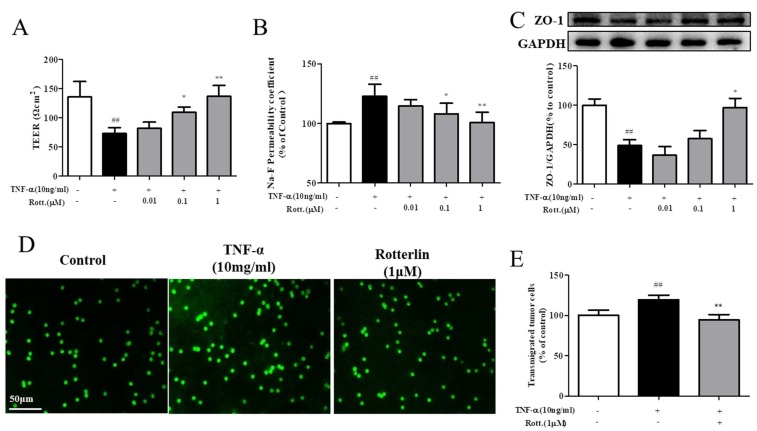
** The PKCδ inhibitor rotterlin inhibited TNF-α-induced endothelial hyperpermeability and transendothelial migration of A549 cells. HUVECs were pretreated with rotterlin (0.01, 0.1, 1 μM) and then exposed to TNF-α for 4 h. (A)&(B)** Effect of rotterlin on TNF-a-induced HUVEC permeability. TEER and Na-F permeability coefficient were determined using a fluorescence multiwall plate reader [Ex(λ) 485 nm; Em(λ) 530 nm]. **(C)** Effect of rotterlin on ZO-1 expression in TNF-a-induced HUVECs. Western blotting analysis revealed that Rotterlin could suppress degradation of ZO-1 in HUVECs induced by TNF-α (10 ng/mL). **(D)&(E)** HUVECs grown to confluence on transwell inserts were activated or not (control) with TNF-α (10 ng/mL). After 4 h, calcein-AM-labeled A549 cells were seeded onto the inserts. After 48 h, melanoma cell migration across HUVEC monolayers was analyzed. Bar graphs show the mean±s.e.m. (n=6 random fields from a duplicate determination). ##*P*<0.01 vs. control group, **P<0.01 vs. model group treated with TNF-α alone.

First, the effects of the PKCδ inhibitor rotterlin (0.01, 0.1, 1 M) on TNF-α-induced endothelial permeability were assessed by TEER and Na-F assays, and the results are shown in Fig. 4A&B. TNF-α stimulation increased endothelial permeability, TEER decreased significantly, and Na-F leakage increased significantly, while rotterlin treatment significantly inhibited endothelial permeability at a concentration of 0.1 μM. Second, we studied the effects of rotterlin (0.01, 0.1, 1 μM) on the TNF-α-induced TJ protein ZO-1 by Western blotting analysis. The results shown that rotterlin could inhibit the TNF-α-induced degradation of ZO-1 in a concentration-dependent manner. Meanwhile, the effect of the PKC δ agonist TNF-α on ZO-1 was investigated. The results shown that ZO-1 degradation could be induced by TNF-α alone, and its function was similar to that of TNF-α, indicating that PKC δ plays an important role in regulating endothelial permeability.

As shown in Fig. 4D&E, after TNF-α stimulation for 4 h, the endothelial cell permeability increased, and the transendothelial migration of tumor cells of A549 cells increased significantly. The PKC δ inhibitor rotterlin (1 μM) could inhibit metastasis, and the effect was significant.

### Effect of C1 on TNF-α-induced Src activity

**Figure 5:**
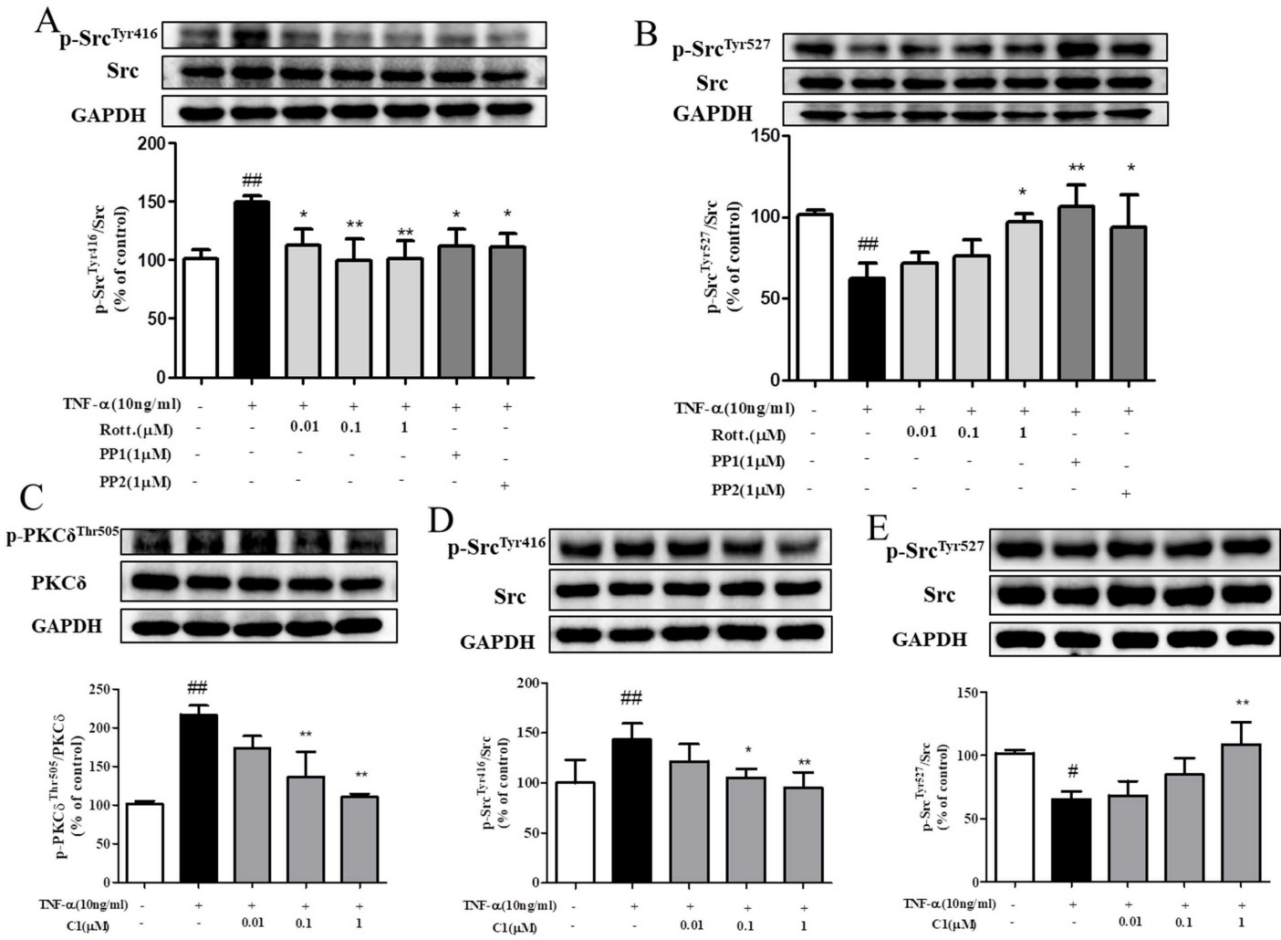
** C1 inhibited activation of protein kinase PKCδ and Src in HUVECs induced by TNF-α. (A)&(B)** HUVECs pretreated with rotterlin (0.01, 0.1, 1 μM) or PP1/PP2 followed by TNF-α for 4 h. Western blotting analysis revealed that rotterlin (1 μM) significantly inhibited activation of Src protein kinase. **(C)** Effect of C1 on activation of PKCδ in HUVECs after TNF-α stimulation for 15 min.** (D)&(E)** Effect of C1 on activation of Src in HUVECs stimulated by TNF-α for 4 h. The data represent the mean±SD from three experiments.^ #^ *P*<0.05, ^##^ *P*<0.01 vs. Control group; ^*^ *P*<0.05, ^**^ *P*<0.01 νs. the TNF-α group.

The results, as indicated by Fig. 5A & B, shown that stimulation with TNF-α activated the intracellular Src protein kinase via phosphorylation of Src and mediated the downstream reaction. Pretreatment with the PKCδ inhibitor rotterlin (0.01, 0.1, 1 μM) inhibited Src protein kinase activation and blocked the downstream reaction, and the inhibitory effect of rotterlin (1 μM) was comparable to that of the Src protein kinase inhibitor PP1/PP2. Activation of Src protein kinase is regulated by the Tyr416 and Tyr527 residues. The Tyr527 localization of inhibitory Src kinase is mediated by self-phosphorylation, while Tyr416 dephosphorylation and activated Src kinase show the opposite results 27. Analysis of the phosphorylation level of Src kinase at these sites can fully explain the activation state.

As indicated by Fig. 5C, D & E, HUVECs were stimulated by TNF-α, and both PKCδ and Src were activated by phosphorylation and mediated the downstream reaction. Pretreatment with C1 (0.01, 0.1, 1 μM) inhibited the activation of PKC δ and Src protein kinase and then blocked the downstream reaction.

### Effect of C1 on the expression of p-PKCδ and p-Src in nude mice

**Figure 6:**
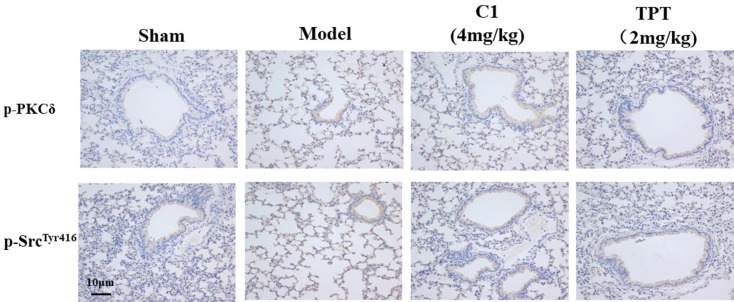
** C1 suppress the expression of p-PKCδ and p-Src in the nude mouse model of experimental pulmonary adenocarcinoma metastasis.** Lung tissue frozen sections were washed with PBS for 5 min. The primary antibodies anti-mouse p-PKCδ (1:200 dilution; Abcam), anti-p-SrcTyr416 (1:200; Abcam) were incubated with tissue sections for 60 min at room temperature. Sections were re-stained with hematoxylin. After dehydration and sealing, sections were observed under a microscope.

As shown in Fig. 6, the p-PKCδ and p-Src protein expression was increased in the model group detected by immunohistochemistry. With the treated by C1(4mg/kg), the expression of p-PKCδ and p-Src reduced in the lung silice(Fig 6A & B). The result shown that C1 could inhibit the expression of p-PKCδ and p-Src in the model.

## Discussion

In this study, we investigated the regulatory effects of C1 on lung cancer and its vascular endothelial barrier function and explored the mechanisms of C1. C1 can inhibit lung cancer in vivo, regulate the level of plasma inflammation in tumor-bearing mice, and protect the pulmonary vascular barrier against injury induced by cancer. We investigated the expression and distribution of the TJ index protein ZO-1 in mouse vascular endothelium and HUVECs and found that C1 could inhibit the degradation and breakage of the ZO-1 protein. Related signaling experiments confirmed that C1 can inhibit TNF-α and activation of PKCδ and Src kinase, thereby reducing endothelial permeability and improving barrier function.

Tumor development is closely related to inflammation. The inflammatory microenvironment of the primary site of the tumor promotes growth, development and metastasis 28. Research has shown that the TNF-α level in cancer patients and other patients is closely related to prognosis 29, 30. High levels of inflammation in tumor-bearing mice can increase vascular permeability, and tumor tissues also secrete many factors to induce the destruction of vascular endothelial integrity 31, 32. Along with the deterioration of other tissues during the aging process, immune system function declines as well 33. Hence, immune surveillance is a key factor in preventing cancer progression, and immunosenescence is another important factor that links tumorigenesis and aging 34. Increased permeability results in leakage of the vascular endothelial cells from the circulation into lung tissues and creates favorable conditions for tumors, while C1 can significantly inhibit the increase in vascular permeability, protect vascular integrity, block tumor cell exudation, and inhibit the metastasis of tumor cells (see Fig. 1). The in vitro experiments in this study examined the effects of the inflammatory factor TNF-α; thus, in nude mice in vivo, plasma TNF-α levels were detected. The results shown that plasma TNF-α increased significantly in the nude model mice, while C1 significantly reduced the increase in plasma TNF-α levels caused by tumor inoculation, and inflammation significantly improved the tumor microenvironment (see Fig. 2D).

Lung cancer is the most frequent cause of cancer-related death worldwide and has a high rate in many elderly patients 35. In cancer metastasis, vascular endothelial cells form the major barrier of tumor cells moving from the tissue to the circulatory system to the tissue 36. The increase in vascular endothelial cell permeability provides favorable conditions for the metastasis of tumor cells, and the close connection of endothelial cells is an important basis for the vascular barrier. TJs are important factors in endothelial integrity. TJs can effectively block cell gaps and form between cell barriers 37. Clinical studies shown that the transcription level of TJ proteins in patients with NSCLC was significantly decreased, and their expression levels were closely related to the prognosis of NSCLC patients. Therefore, by protecting endothelial cell integrity, maintaining TJs of endothelial cells, and inhibiting invasion and metastasis of lung cancer cells, effective intervention in the early stage of lung cancer occurrence and metastasis may be an important direction for future research and development of anti-lung cancer metastasis drugs 38. The determination of transendothelial resistance and the determination of the change in cell culture chamber material leakage rate are used to assess cell permeability; by using a TEER tracer to measure the transfer current for solute ion strength, permeability changes can be recorded in real time. Na-F assays of monolayer cells and macromolecules were also performed 39. In this study, TEER and Na-F were used to observe the effect of C1 on HUVEC permeability induced by TNF-α, and the HUVEC permeability was examined in different assays. The results were reasonable and consistent (shown in Fig. 1). Meanwhile, we used the nude mouse model of experimental pulmonary adenocarcinoma metastasis to study the anti-tumor effect of C1. The results shown that there were significantly fewer tumor nodules in the C1 and TPT groups than the model group, which demonstrated that C1 can inhibit the lung tumors in vivo (see Fig. 2).

Intercellular TJs are a transmembrane protein complex composed of transmembrane proteins (Occludin, Claudins polygene family, JAM) and cytoplasmic proteins (ZO-1 and others) 40. The ZO-1 protein is the most important protein in the cytoplasm, constitutes an important component of the TJs, and connects to the main cell body to act as intermediate transmembrane protein and part of the actin cytoskeleton. Functional and structural changes can cause dissociation of TJs and increased intercellular space, thereby increasing the vascular permeability 12. The expression and structural changes of ZO-1 involve a series of complex intracellular signal transduction pathways, including tyrosine kinase Src, protein kinase C, Ca^2+^, G protein, cAMP and other signaling pathways 41. When endothelial cells are stimulated by TNF-α, the permeability of endothelial cells increases and the ZO-1 protein is degraded. Some studies have shown that Src kinase activation is involved in the formation of TNF-α-induced vascular inflammation and plays an important role in the endothelial cell barrier function 42. Thus, the TJ protein ZO-1 was significantly degraded after TNF-α stimulation, and pretreatment with C1 could significantly inhibit the degradation of ZO-1 in a concentration-dependent manner (see Fig. 3A & B). Compared with the control group, the model group shown decreased ZO-1 expression. C1 and TPT administration could significantly improve pulmonary vascular permeability and increase the expression of ZO-1 protein, indicating that the anti-tumor effect of C1 was related to its protection of pulmonary vascular integrity (see Fig. 3C & D).

We also found that the positive control drug TPT significantly regulates the expression of ZO-1, and this regulation is related to the inhibition of PKCδ activation (see Fig. 3C & D). TPT has a slight clinical effect in the treatment of small cell lung cancer. This drug binds to the topoisomerase I-DNA complex, preventing the repair of single-stranded DNA after reversible disruption induced by topoisomerase I, resulting in cell death 43, 44. This study found that TPT inhibited the destruction of the lung vascular barrier induced by cancer and provides a new clue for its mechanism.

PKCδ can be activated through a Src-dependent mechanism. Src regulation of PKCδ was also reported 21. However, PKCδ has little effect on Src, and this has not been reported in vascular endothelial cells. To further elucidate the possible molecular mechanism of C1 in regulating TNF-α-induced barrier function of HUVECs, we investigated the effect of the PKCδ inhibitor rottlerin on TNF-α-induced endothelial permeability, TJ protein ZO-1 degradation, A549 transendothelial migration and activation of Src kinase. TEER, Na-F leakage, HUVECs-A549 cell co-culture and Western blotting results shown that rottlerin can significantly inhibit TNF-α-induced HUVEC permeability, A549 transendothelial migration, and activation of Src kinase in a concentration-dependent manner (see Fig. 4).

Clinical studies have shown that in NSCLC tumor tissues, the protein expression of the kinase PKCδ is increased, and rottlerin can effectively induce apoptosis of tumor cells 18. Moreover, with the inflammatory factor TNF-δ or IL-1β, PKCβ participates in the increase of endothelial cell permeability and the degradation of the closely related connexin ZO-1 19, 45, 46. Therefore, interfering with protein kinase PKCδ and Src, regulating the close connection of endothelial cells and protecting endothelial barrier function may be an effective strategy to inhibit tumor metastasis. Other studies have shown that PKCδ is involved in inflammatory factor-mediated endothelial inflammatory models 19. In addition, C1 (0.01, 0.1, 1 μM) significantly inhibited TNF-α-induced phosphorylation of the PKCδ and Src kinase proteins (see Fig. 5).

In traditional Chinese medicine, "*Ophiopogon japonicus*" was first used in Shennong Ben Cao Jing. It is commonly used in traditional Chinese medicine. Its medicinal properties are sweet, bitter and cold. This medicine is believed to moisten the lungs, promote fluid, nourish the yin and clear away heat. Modern pharmacological research has shown that *O. japonicus* has anti-inflammatory and anti-tumor effects and protects against endothelial damage 47-49. The main chemical components of *Ophiopogon* are steroid saponins, isoflavones, phenolic acids, polysaccharides and others. A previous study by our group shown that the saponins from Ruscogin were the main active components of anti-inflammatory and anti-tumor drugs. Ophiopogonin DT-13 was reported to be involved in regulating TNF-α-induced endothelial inflammation, regulating the activity of non-muscle myosin IIA under hypoxia, and inhibiting the metastasis of 95D cells in lung cancer 23, 50, 51. However, whether other saponins in *O. japonicus* can exhibit stronger anti-inflammatory effects and protect endothelial integrity and tumor metastasis activity is not clear. Whether they can play a protective role by regulating the activity of PKCδ and Src is also unknown. In this study, we used a nude mouse model of experimental pulmonary adenocarcinoma metastasis to study the anti-metastasis effects of C1, a new chemical compound isolated from *O. japonicus*. The results shown that the tumor nodules in the C1 and TPT groups were significantly fewer than those in the model group, which demonstrated that C1 can inhibit the metastasis of lung tumors in vivo. At the same time, the effects of C1 on pulmonary vascular permeability and endothelial TJs were investigated. Compared with the control group, the model group had significantly higher pulmonary vascular permeability and significantly decreased ZO-1 expression. C1 and TPT administration could significantly improve pulmonary vascular permeability and increase the expression of ZO-1 protein, indicating that the anti-metastasis effect of C1 was related to its protection of pulmonary vascular integrity. In this study, we investigated the effect of C1 on the TJ proteins of HUVECs using immunohistochemistry and Western blotting. The results shown that the TJ protein ZO-1 was significantly degraded after TNF-α stimulation, and pretreatment with C1 could significantly inhibit the degradation of ZO-1 in a concentration-dependent manner.

We found that C1 had an obvious effect on TNF-α-induced endothelial cell barrier damage and verified its anti-tumor effect on lung cancer in vitro and in vivo. We also demonstrated that the inhibition of PKCδ and Src is one important mechanism underlying the protective effects of C1 on vascular endothelial integrity, which may explain other activities of *O. japonicus*.

Therefore, we used vascular endothelial cells to assess the changes in endothelial permeability and TJ function and structure. Our findings lay the foundation for the discovery of the new drugs based on *Ophiopogon* saponins. Meanwhile, our results provide experimental evidence demonstrating the role of the vascular endothelium in tumor metastasis and may contribute to the development of anti-tumor metastasis drugs from effective components of traditional Chinese medicine. Therefore, starting from traditional Chinese medicine theory and using modern medical research methods, researchers should search for highly active drugs to inhibit metastasis of lung cancer from *O. japonicus*, which may contribute to the research and treatment of tumors and the rational application of traditional Chinese medicine. Despite the above research results, there are still some limitations of this study. Further research is needed to determine whether the regulation of endothelial function by ophiopogonin A1 and C1 plays a direct role in PKC delta or Src. Are there other key proteins upstream? In a whole animal model, how does C1 regulate the level of plasma inflammation in tumor-bearing mice? Is there a direct correlation between the protective effect of *O. japonicus* saponins and the level of plasma inflammation? The above problems were studied by RNA interference and gene knockout to help elucidate the molecular mechanism of *O. japonicus* saponins A1 and C1 in the prevention and treatment of lung cancer metastasis and lay the foundation for its clinical development.

## Supplementary Material

Supplementary table S1.

## Abbreviations

BSA: Bovine serum albumin
EBA: Evan's blue-labelled albumin
ECL: Enhanced chemiluminescence
FBS: Fetal bovine serum
HRP: Horse radish peroxidase
HUVECs: Human umbilical vein endothelial cells
Na-F: Fluorescein sodium
NSCLC: Non-small cell lung cancer
PBS: Phosphate buffer salts
PKC: Protein kinase C
PVDF: Polyvinylidene fluoride
SDS: Sodium dodecylsulfate
TEER: Trans-endothelial electrical resistant
TJs: Tight junctions
TNF-α: Tumor necrosis factor-α
TPT: Topotecan hydrochloride
Tris: hydroxymethyl aminomethane
