# Supplementary material for: Ophiopogon Saponin C1 Inhibits Lung Tumors by Stabilizing Endothelium Permeability via Inhibition of PKCδ
Source: Int J Biol Sci. 2020 Jan 1;16(3):396–407. doi: 10.7150/ijbs.34978 (PMC6990896; doi:10.7150/ijbs.34978)

Supplement Figure 1. C1 ameliorated TNF-α-induced endothelial hyperpermeability and **transendothelial migration of 95D cells.** EC permeability was measured using a Millicell-ERS voltohmmeter. HUVECs were pretreated with various concentrations of C1 (0.01, 0.1 and 1 μM) for 1 h followed by TNF-α (10 ng/mL) stimulation for 4 h. **(A)** The transendothelial permeability was assessed by TEER. **(B)** The transendothelial permeability was assessed using the paracellular transport marker (Na-F) permeability coefficient, which was measured using a fluorescence multiwall plate reader [Ex (λ) 485 nm; Em (λ) 530 nm]. **(C)&(D)** HUVECs grown to confluence on transwell inserts were activated or not (control) with TNF-a (10 ng/mL). After 4 h, calcein-AM-labeled 95D cells were seeded onto the inserts. After 48 h, melanoma cell migration across HUVEC monolayers was analyzed. Bar=50 μm. The data represent the mean±SD from three experiments. ##*P*<0.01 vs. the control group; **P*<0.05, ***P*<0.01 vs. the TNF-α group.

(see original image in file pdf)

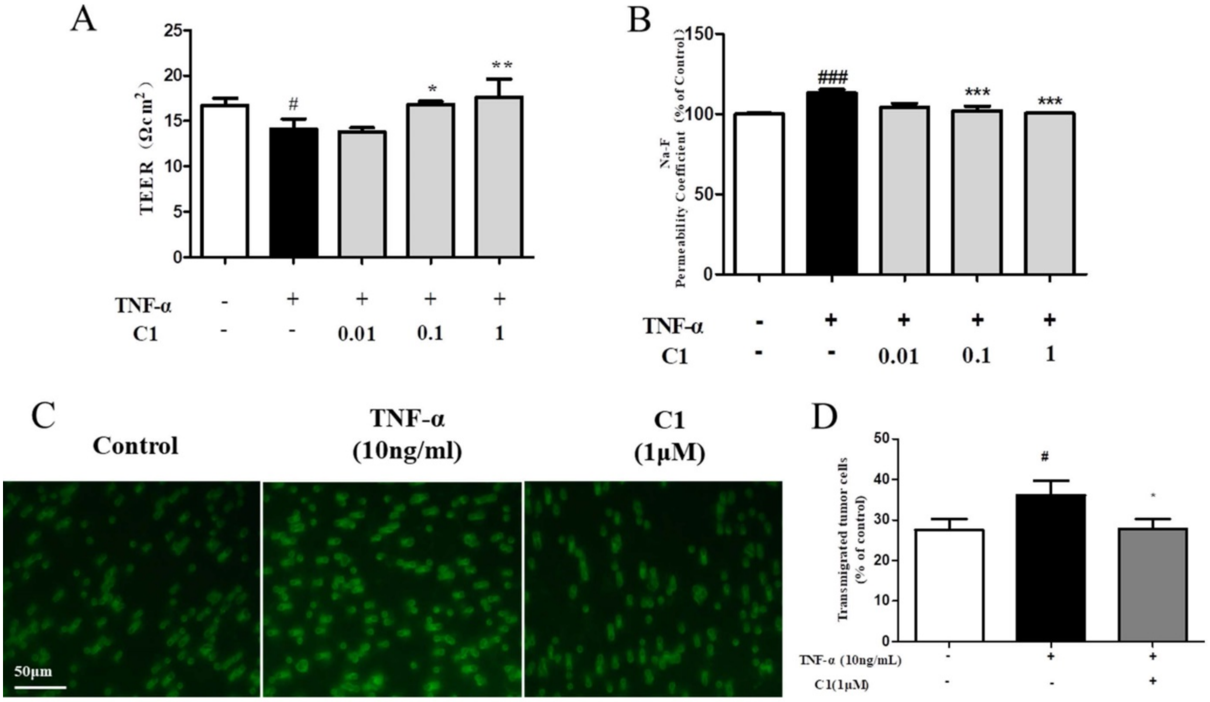

Supplement: Supplementary file 1 — Supplementary table S1. [file ijbsv16p0396s1.pdf]
